# A new patient-specific overformed anatomical implant design method to reconstruct dysplastic femur trochlea

**DOI:** 10.1038/s41598-023-30341-4

**Published:** 2023-02-24

**Authors:** Yetkin Öztürk, Murat Ayazoğlu, Çağrı Öztürk, Atakan Arabacı, Nuri Solak, Serhat Özsoy

**Affiliations:** 1 Molecular Biology and Genetics Department, Science and Literature Faculty, Istanbul Technical University, Maslak, 34469 Istanbul, Turkey; 2 Faculty of Manufacturing Engineering, Istanbul Technical University, Gumussuyu, 34437 Istanbul, Turkey; 3 Metallurgical and Materials Engineering Department, Chemical, and Metallurgical Engineering Faculty, Istanbul Technical University, Maslak, 34469 Istanbul, Turkey; 4 Surgery Department, Veterinary Faculty, Istanbul University-Cerrahpasa, Buyukcekmece, 34500 Istanbul, Turkey

**Keywords:** Biotechnology, Biomaterials, Biomedical engineering

## Abstract

Patellar luxation with condylar defect is a challenging situation for reconstruction in humans. Patella reluxation, cartilage damage and pain are the most common complications. This study aims to present a new patient specific method of overformed implant design and clinical implantation that prevents luxation of patella without damaging the cartilage in a dog. Design processes are Computer Tomography, Computer Assisted Design, rapid prototyping of the bone replica, creation of the implant with surgeon’s haptic knowledge on the bone replica, 3D printing of the implant and clinical application. The implant was fully seated on the bone. Patella reluxation or implant-related bone problem was not observed 80 days after the operation. However, before the implant application, there were soft tissue problems due to previous surgeries. Three-point bending test and finite element analysis were performed to determine the biomechanical safety of the implant. The stress acting on the implant was below the biomechanical limits of the implant. More cases with long-term follow-up are needed to confirm the success of this method in patellar luxation. Compared with trochlear sulcoplasty and total knee replacement, there was no cartilage damage done by surgeons with this method, and the implant keeps the patella functionally in sulcus. This is a promising multidisciplinary method that can be applied to any part of the bone and can solve some orthopaedic problems with surgeon’s haptic knowledge.

## Introduction

Recurrent patellar instability is a problematic condition that can cause pain, patellofemoral osteoarthritis, and decreased knee movement. Many factors affect patellar dislocation. These are abnormal structure of the patella and trochlea, abnormal limb alignment and incorrect ligament and muscle interactions^1,2^. Abnormal shape and depth of the trochlear groove is called trochlear dysplasia (TD). There is an insufficient bony restriction of the femoral condyle which mal-tracks the patella during knee flexion. The 4-grade Dejour classification is used to identify the grade of TD^3,4^. When the TD degree is high, treatment is based on trochleoplasty combined with other surgical corrections such as medial/lateral releasing, tuberositas tibia transposition. Trochleoplasty is to change the shape of the articular surface of the distal femur by osteotomy of the trochlea. These operations are successful in keeping the patella in the groove. However, the risk of cartilage damage, arthritis and pain is high^5,6^. Because of these complications, a new method will be useful to hold the patella in place^7^.

Additive manufacturing technology helps to fabricate custom-fit, patient-specific orthopaedic models and prosthetic implants for any part of the body^8^. Patient-specific implants are very popular for solving anatomical and pathological bone problems. They are made particular to a patient’s specific anatomy^9^. Three-dimensional imaging methods such as magnetic resonance imaging (MRI) or computer tomography (CT) can be used to get patient-specific bone data. These help to obtain fast, high resolution three-dimensional (3D) views of relevant parts of the body. A personalized implant design is made by adapting the geometry of the implant to the bone using computer aided design (CAD). This creates a 3D solid model and then can be printed by a 3D printer. It is beneficial to use 3D printed implants in orthopaedics. They have the potential to create better solutions for complicated bone disorders^10–13^. However, reconstructing the normal anatomy may not always be functional in multifactorial problems^4,14,15^.

We think that a new extra-anatomic shape of a bone structure may solve some problems under special circumstances. In this study, we tried to heal the chronic patellar luxation of a dog with a highly dysplastic femoral trochlea. We made an overformed patient-specific implant using the 3D printed bone replica, a self-hardening putty, and the surgeon’s haptic anatomical knowledge.

## Material and methods

### Clinical data and preoperative planning

**Figure 1:**
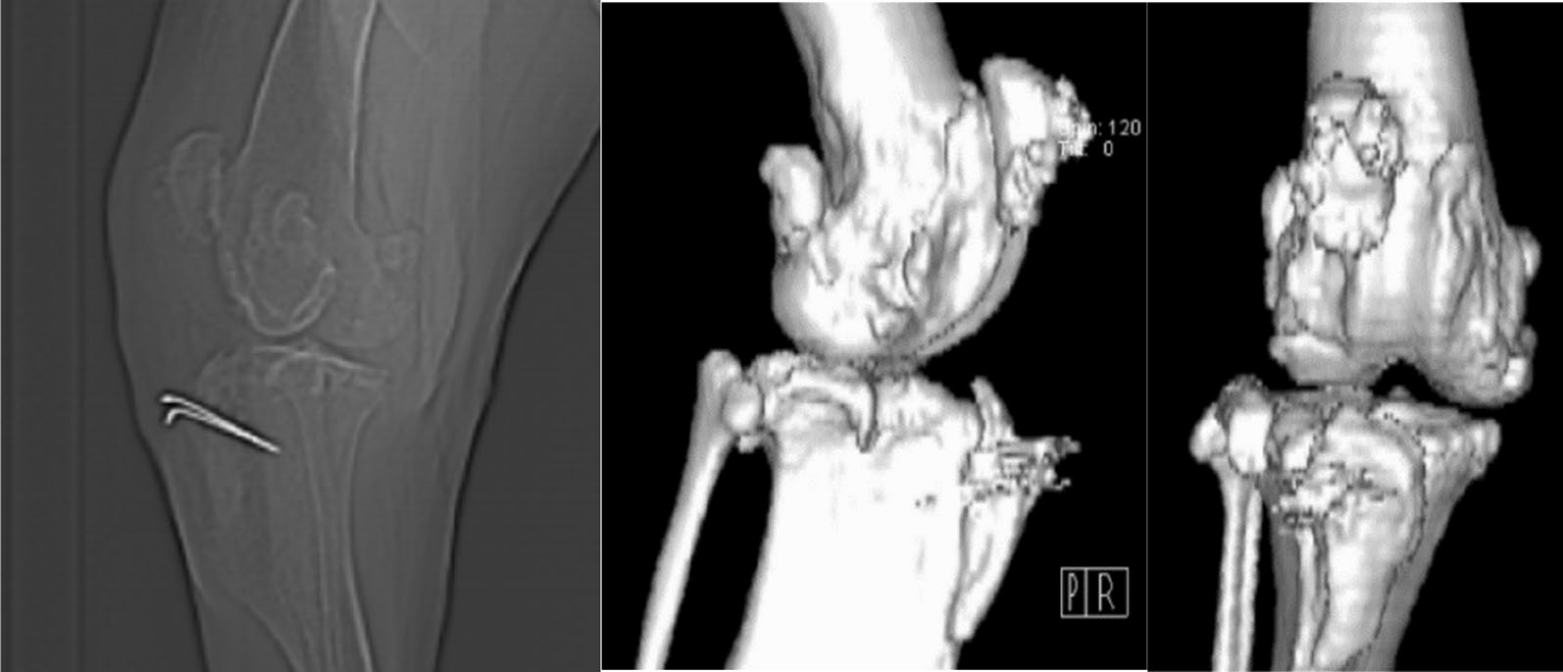
Preoperative CT views of the dog. There is contact between femur and patella. Patella could luxate easily to the lateral side of the femur.

A 3-year-old female, 46 kg Mastiff dog was diagnosed with fourth-degree lateral patellar luxation. The dog had two operations before. These were capsulectomy, capsulorrhaphy, medial releasing, sulcoplasty and tibial tuberosity transposition. Together with a fistule problem patella still luxated. The CT scan of the dog’s femur was performed with a Siemens Spirit Dual Slice Refurbished CT scanner (SBM Healthcare, India) and 2.0 mm slice thickness (Fig. 1). The 3D model of distal femur was constructed from CT data for a better understanding the deformed anatomy of the bone and implant fabrication planning to keep patella in situ. It was planned to add an over-formed implant to the deformed lateral condylus of the femur that could prevent the dislocation of the patella. Therefore, a self-hardening putty was decided to be shaped by a veterinarian surgeon’s hand. After that it was planned to print the implant by using 3D scanner and 3D printer with biocompatible material. An Informed Consent was signed by owner of the dog.

### Design criteria and method for the implant

According to the location of the lateral femoral condyle, implant to be fixed to the defected area needs some criteria^16^. These are safety, overformed shape, rapport with bone and soft tissues, and functionality. The implant material was polyamide to be biocompatible material. Overformed shape was needed to overcome extreme pulling forces of quadriceps muscles to the lateral side because of chronic developmental deformity. The implant’s lateral trochlear ridge was intended to be higher than the medial trochlear ridge but had to stay under the capsula. It should not prevent functionality of the knee joint during extension and flexion, and should ensure that patella stays in situ.

**Figure 2:**
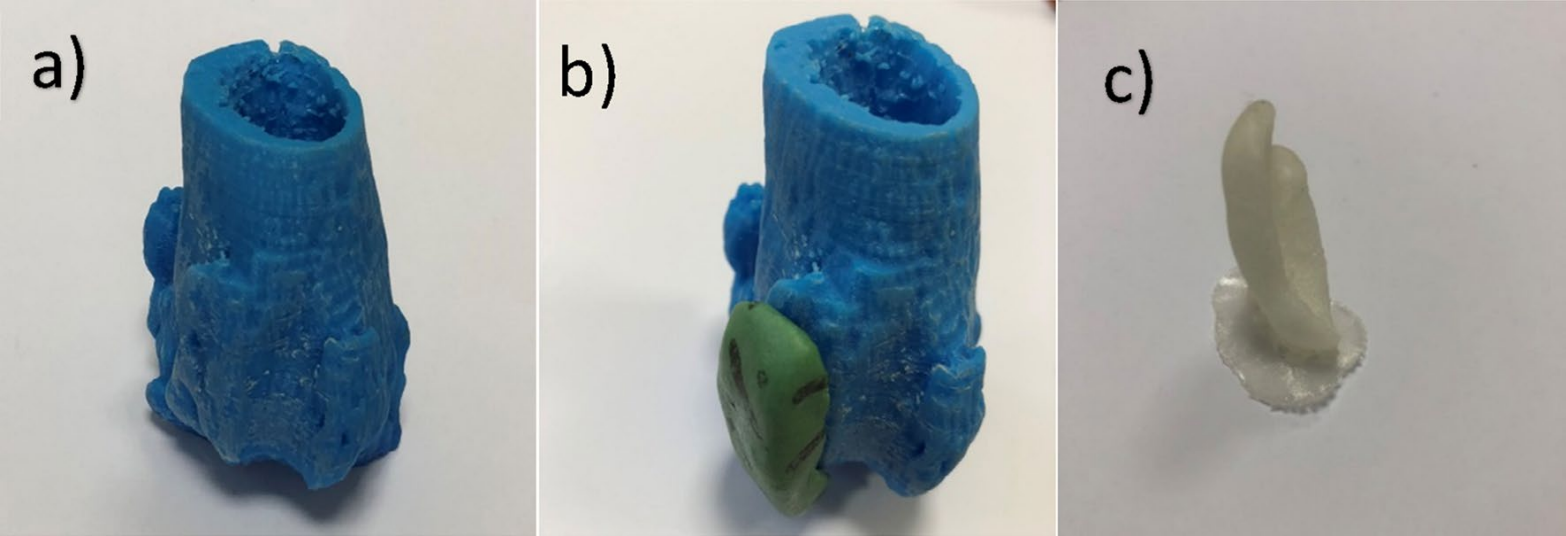
Bone replica created with 3D printer by ABS material (**a**), self-hardening putty (green) which was the implant replica, made by surgeon according to haptic knowledge and surgical experiences (**b**), polyamide implant printed after the implant replica scanned with 3D scanner and stl data was used in 3D printer (**c**).

The design workflow of overformed implant begins with detecting and printing the anatomic shape of distal femur by 3D printer using Acrylonitrile Butadiene Styrene (ABS) plastic material which was replica of original distal femur. To make overformed implant (not to keep the normal anatomic shape) a self-hardening putty (Sun-Fix, Germany) was used by surgeon. Surgeon used this putty directly on the defected side of the replica with hands and created the overformed implant over the replica. Surgeon decided the margins and size of the implant according to haptic knowledge and surgical experiences. Margins of the implant were created at sufficient height so that the patella does not dislocate again. Once the paste had set, the replica and putty (implant replica) were separated. The shape of the overformed implant replica was transferred to computer by 3D scanning technology. Transferred data was used to print the overformed implant with polyamide material by 3D printer. The outer surface of the implant was rubbed with an emery. Bone replica, implant replica and implant are shown in Fig. 2.

### Fabrication of the implant

**Figure 3:**
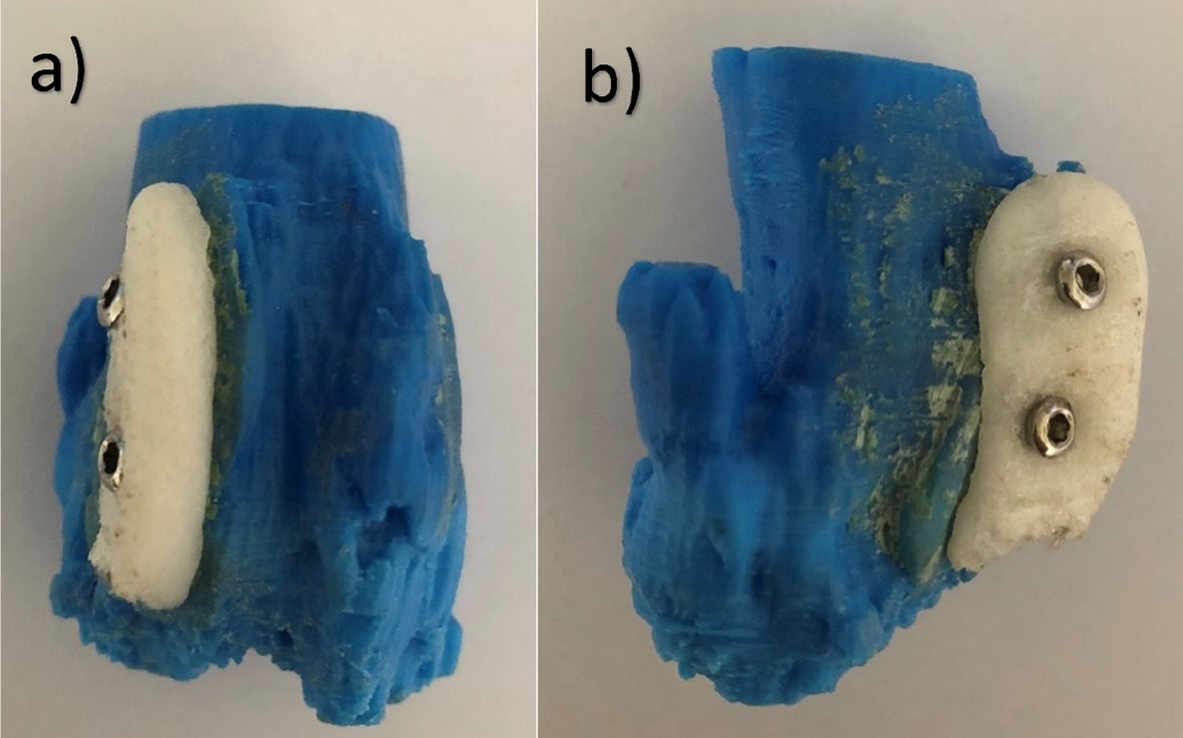
After the implant was printed the proper sitting was checked on the bone replica. Anterio-posterior view (**a**) and lateral view (**b**) of the implant on the bone replica. Inner surface of the implant fits very well with surface of the bone replica.

The replica of the distal femur was printed by fused deposition modelling machine (Ultimaker 2+, Netherland) using ABS material. The nozzle diameter of the printer was 0.4 mm, the printing speed was 50 mm/s, the printing thickness was 0.1 mm and the nozzle temperature was 260 °C. The amount of self-hardening putty (Sun-Fix, Germany) was determined by surgeon. It hardened approximately 30 min after it was mixed. It was wiggled on 20, 25 and 30 min. The putty was removed from the replica. The shape of the dried putty is obtained by 3D scanner (Artec Space Spider, Santa Clara, CA) in stereolithography (STL) format files. It is printed with 3D printer (Ultimaker 2+, Netherland) using polyamide material. The implant was produced with its supports. After the supports were removed with pliers, the outer surface was sanded with 800 and 1200 grit sandpaper. Correct fit of the inside of the implant was checked on the replica (Fig. 3). When the implant and bone replica are touched by hand, there should be no movement between them. Different sizes of implants can be produced to ensure that the knee joint capsule is sutured without any problem in surgery. We have produced three types of implant. Implant samples were sterilized by an autoclave at 121 °C and 1.5 atm pressure for 20 min.

### Biomechanical analysis of the implant (three-point bending test)

Three-point bending test was performed for biomechanical analysis. Rectangular beam shaped specimens were used for the test because the shape of the implant was irregular, and it could cause inaccurate measurement. The beam shaped specimens were supported at its ends. A point force was applied on the middle of the specimens until the fracture occurs using three-point bending machine (Autograph AGS-J, Shimadzu, China). The machine with maximum load of up to 1 kN, air temperature of 25 °C and attached with high accuracy extensometer. All the five specimens were printed by 3D printer with polyamide material. Bar geometry has been selected for three-point bending test. The sample dimensions were 42 mm, 6,5 mm, 2 mm in compliance with ASTM D790 standards. Implant’s and sample’s thickness and length values were kept same. Printing properties were same as the fabrication of the implant using Ultimaker 2+.

### Finite element analysis

In order to simulate the mechanical performance of the implant with a specific geometry, finite element method (FEM) analysis was carried out by using a commercial computational solver, ANSYS 2021 R1. CT scan (Digital Imaging and Communications in Medicine) images of the femur and implant were converted into solid objects by using an open source engineering software FreeCAD. 3D solid models exported from FreeCAD was assembled by SolidWorks 2019. For the FEM analysis, 3D model was exported to ANSYS Workbench and material properties of each component in the model was specified. Surface-to-surface contact is both contact and target surfaces. The material properties of the implant were taken from three-point bending test. Worst case was assumed, and material property values were taken as lower than the trabecular and cortical bone values in the literature, and calculations were made (Table 1)^17,18^.

**Table 1:** Material properties of bone, implant and stainless steel used in the finite element analysis. Bone properties were defined lower than the original strengths to predict worse conditions for the implant in the operation. Implant properties were defined according to three-point bending test.

Components	Elastic modulus (GPa)	Poisson’s ratio	Yield strength (MPa)
Bone	2.1	0.3	135
Polyamide implant	1.05	0.38	16.93
Stainless steel screws	193	0.31	207

Even though the bone is a natural composite material, mechanical properties of the bone is assumed to be isotropic^19^. Approximately 16.56 kg, which is 36% of the body weight^20^ will pass through the limb for a dog weighing 46 kg. A 20 kg of force was applied from the top section of the bone and the lower section of the bone was defined as the fixed support to keep the rigidity of the model and ensure required conditions for static analysis. The number of elements in the mesh was optimized to achieve high quality and proper results. Therefore, a tetrahedral mesh type with a total number of 240,020 elements and 410,815 nodes were used for the analysis. The mesh size was 0.5 mm.

### Surgical implementation

**Figure 4:**
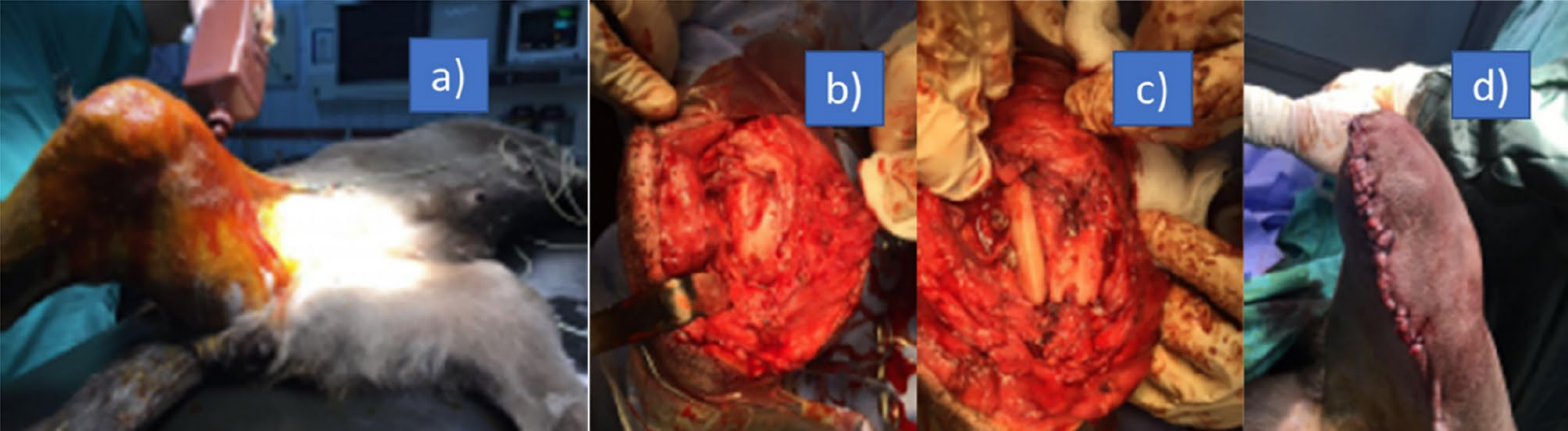
Preoperative photograph (**a**), deformed lateral femoral condylus (**b**), overformed polyamide patient specific implant fixation with two 3,5 mm cortical screws (**c**), postoperative photograph of the dog (**d**).

The study is approved by Istanbul University-Cerrahpasa Veterinary Faculty Ethical Committee. Operation carried out in accordance with relevant guidelines and regulations. All the procedure was performed conformed to the ARRIVE guidelines. Owner consent for participate and consent for publication were obtained from the owner of the dog. 25 mg/kg Ceftriaxone (Unacefin, Yavuz İlaç, Turkey) was given intravenously (IV) as preoperative antibiotic and continued for 1 week postoperatively. 0.1 mg/kg (IV) Meloxicam (Metacam, Boehringer, USA) was used preoperatively and continued for 4 days postoperatively. 1 mg/kg (IV) Xylazine Hydrochloride (Bayer, Germany) was given for premedication and 5 mg/kg Ketamine Hydrochloride (Pfizer, New York) was used IV for induction of the anesthesia. The dog was intubated and isoflurane (Isoflurane, Piramal Critical Care Inc. US) was used for the continuation of anesthesia. The dog monitored during the anesthesia. A craniolateral skin incision was made near patella. Subcutaneous tissue was incised along the same line. Incision to lateral retinaculum and joint capsule was made to expose the joint. Implant was fixed to the lateral side of the condylus with two cortical screws (Fig. 4). They were stainless steel, 3,5 mm thread diameter (fully threaded), 28 mm long cortical screws. Soft tissues were sutured with Poly Lactic-co-Glycolic Acid (PLGA) (Pegalak, Doğsan, Turkey) and skin sutured with Polypropylene (Prolene, Ethicon, US). The dog was hospitalized 80 days at surgery department. Radiographs were examined and walking status were observed.

## Results

### Biomechanical tests

#### Three-point bending test

**Figure 5:**
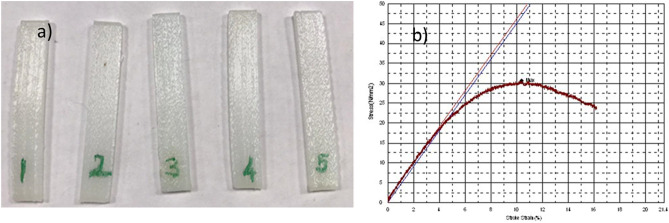
Rectangular beam shaped specimens for three-point bending test (**a**). Stress–strain curve of the sample 1 (**b**).

The beam shaped specimens and the tensile stress–strain curve of the beam shaped specimen 1 is shown in Fig. 5 and all the data of the five specimens are represented in Table 2. The average of the yield strength of the samples is 16.12 MPa and the average of maximum tensile strength is 26.27 MPa.

**Table 2:** Three-point bending test results.

Sample	Maximum tensile strength (N/mm^2^)	Yield strength (0.2%) (N/mm^2^)
1	30.61	18.67
2	24.03	14.66
3	26.32	16.45
4	24.96	15.52
5	25.46	15.3
Average	26.27 (± 8%)	16.12 (± 8%)

#### Finite element analysis

**Figure 6:**
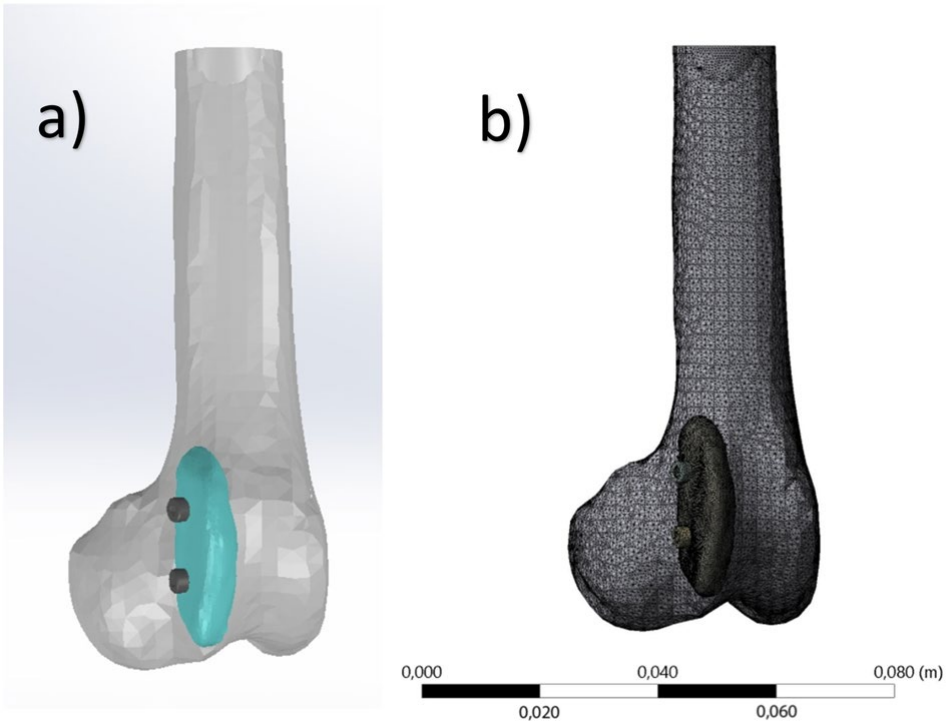
The 3D model (**a**) and its meshed image (**b**).

**Figure 7:**
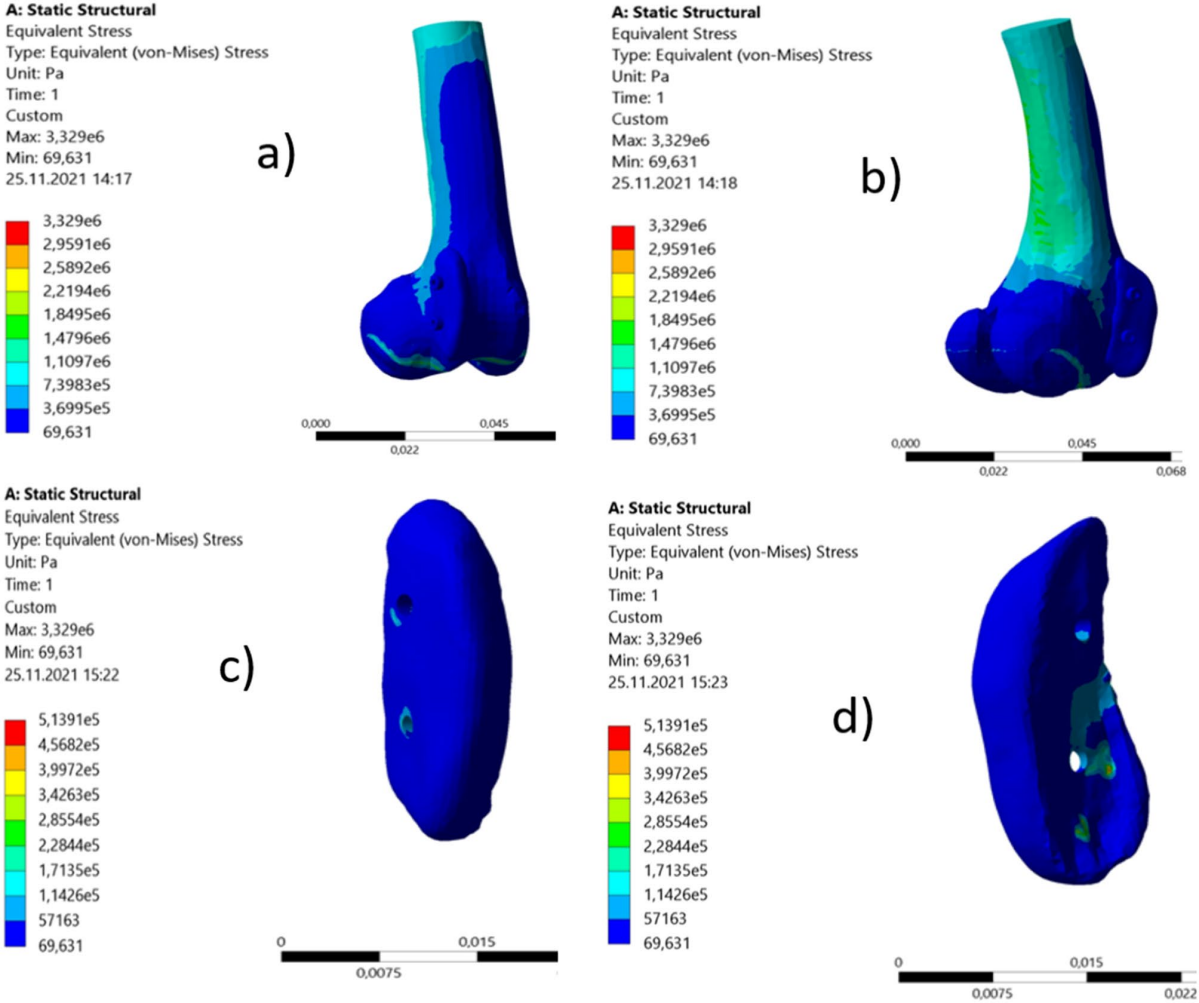
The stress distributions on the bone model and implant. Front view (**a**) and backside (**b**) of the bone model, outer surface of the implant (**c**) and inner surface of the implant (**d**).

Under the loading conditions mentioned before, the maximum equivalent stress is observed around the fixed implant on the lower section of the bone and calculated as 3.29 MPa. The 3D model and its meshed image are shown in Fig. 6. The stress distributions on the model and implant are shown in Fig. 7. Considering the implant, the maximum equivalent stress is calculated as 0.5 MPa.

### Implant application and postoperative evaluation

**Figure 8:**
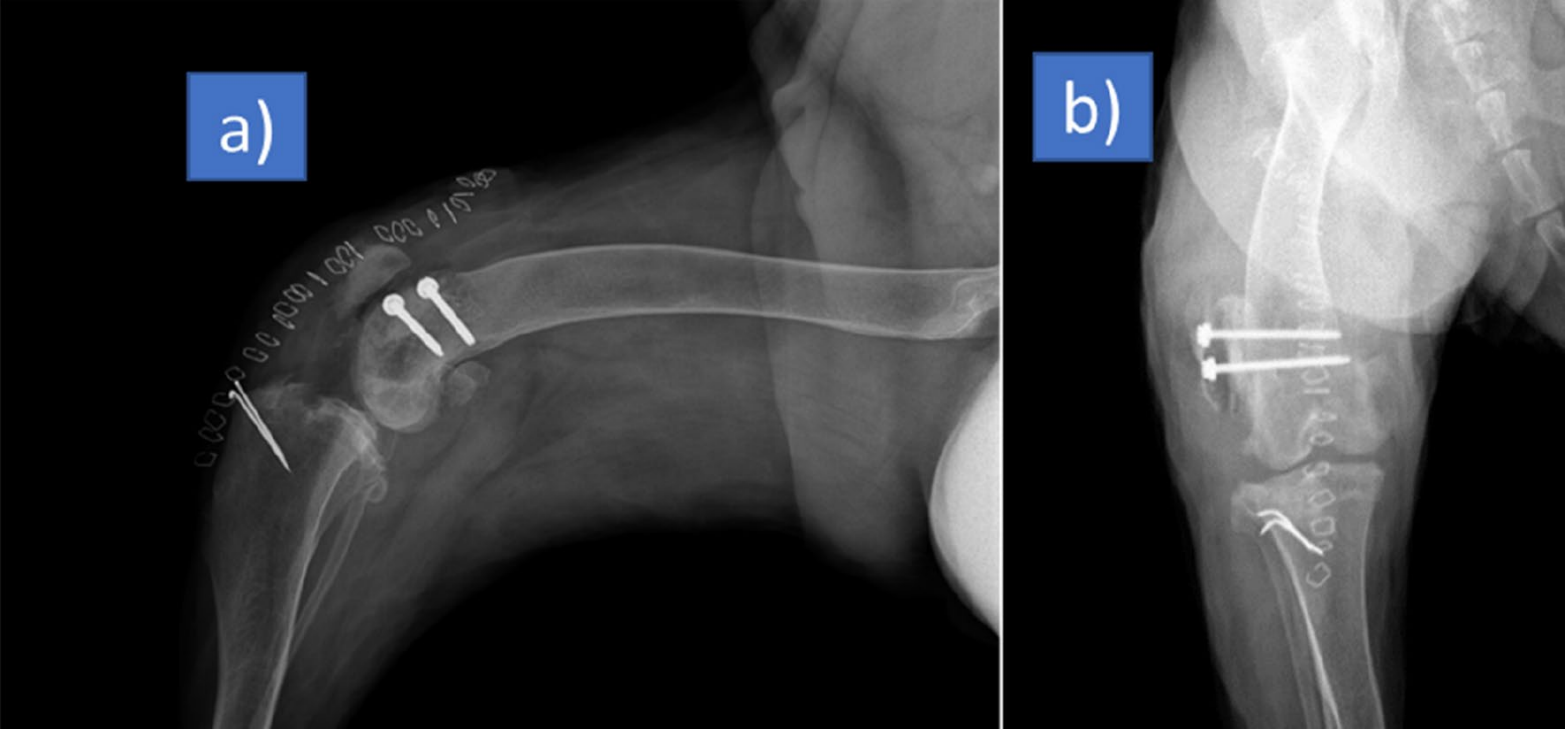
Postoperative rontgen film of the dog. There is no contact between the patella and femur on the lateral view (**a**), patella is on dorsal but slightly lateral side on anterior posterior view (**b**). However, it did not luxate to lateral side spontaneously or with hand. It was stable. The pins on the tibia were applied on previous operations.

**Figure 9:**
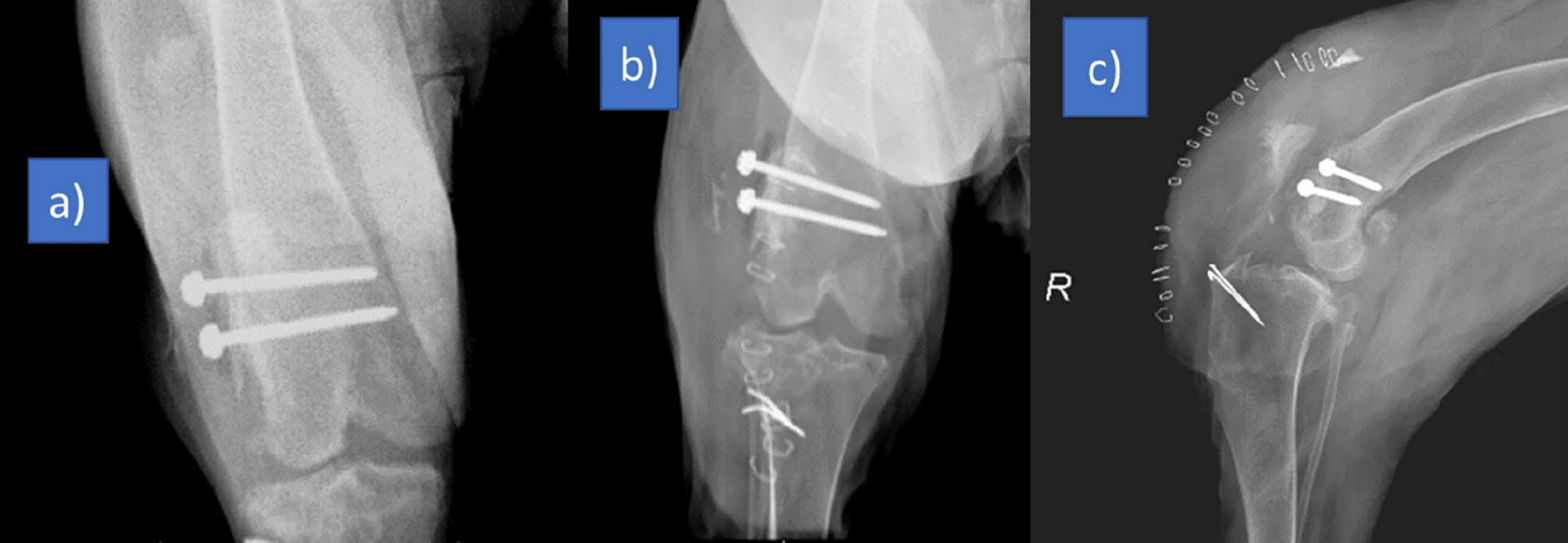
Postoperative anterior–posterior radiograph of the femur on 50 days (**a**), posterior–anterior (**b**) and lateral (**c**) radiograph on the 80 days. Both on 50 and 80 days there were no implant movement and implant-bone reaction. Patella did not luxate. However, there was a fistule problem before the operation. It progressed and made inflammation. The swelling can be seen on lateral radiograph (**c**).

The implant fitted perfectly to the bone surface. There was no movement between the contact surfaces of the implant and the lateral femoral condyle. Two 3.5 mm cortical screws fixed the implant tightly. After the implantation, capsula and retinaculum sutured without difficulty. Patella was on the dorsal of the trochlea but slightly laterally without creating a patellar luxation spontaneously or manually as just after seen in the postoperative rontgen film (Fig. 8). There was no implant failure after 50 and 80 days according to the rontgen controls. According to the preoperative rontgen films and tomography scans patella was no longer lateral to the condyle on the 50 and 80 days (Fig. 9). Preexisting fistule problem did not heal and reoperated for fistule again on 80 days. Patella was in situ. However, the dog walked with a limp. There was no bone implant reaction. Inflammation signs around quadriceps muscles and knee have been detected.

## Discussion

There are many polymers used in 3D implant printing. Structure and surface compatibilities have to meet between the implant and the patient. Correct choice minimizes the immune response^21,22^. Polyamide is one of them which is a biocompatible material. There may be free space inside the implant according to printing protocol^23,24^. In the screwing process, the screw heads pushed the implant inward slightly and stopped. The cortical screws fixed the implant to the bone very tightly. Osteconductive implants could also be used in bone defects^25^. But we wanted to observe direct mechanical effect of our implant. These features can be combined in future studies. In evaluation of sterilization, if there was a microbial growth in the yeast extract peptone dextrose, there should be turbidity and colour change after 3 days^26,27^. Absence of turbidity and discoloration after 11 days of incubation indicated no microbial growth in the overformed implant after sterilization. There was no loosening or breakage of the sterilized implant, which was successfully screwed into the condyle in the operation. It had no further screw loosening or implant failure problem afterwards.

We did not use the literature data for material properties of polyamide because when a material is produced with a 3D printer, it effects the material properties. Sufficient bonding between material strands do not occur perfectly during the deposition process. Effects of porous structures or voids on the material properties of a product are also important. The performance is reduced^28^. Stress/strain results of the polyamide 3D printed material were worse than bulk polyamide material^29^. However, they were approximately same with other studies performed with 3D printers^30,31^. Approximately 16.56 kg (36% of the body weight for pelvic limb^20^) will pass through the limb for a dog weighing 46 kg. We applied 20 kg in the finite element analysis to the femur bone.

Mechanical properties of the bone is assumed to be isotropic, homogenous, and linear elastic to simplify the problem^19^. Isotropic material models can be considered sufficient for the femur^32,33^. The von Mises stress is often used to determine whether an isotropic and flexible sample will yield after applied loading conditions^34^. Maximum equivalent stress on the implant was calculated as 0.5 MPa according to the finite element analysis. The average yield strength was 16.12 MPa. It can be concluded that the maximum stress calculated on the model is much lower than the mechanical limits of the implant under the given loading conditions. Under a 20 kg of loading, the location at the lower section of the bone (lateral distal femur) is safe for implementation of the overformed polyamide implant. The implant was applied on the lateral side of the femur. It was not in contact with the articular surface of the condyle, through which most of the body weight passed. This is also another reason that makes it safer against body weight.

There are patient, implant and surgeon factors that affect the outcome of the surgery. The degree of the orthopaedic problem, the experience of surgeon and the functionality of the implant are important to achieve the targeted result^35^. Perfect anatomical implant shape is critical to have the best implant surgery results^36^. Chronic fourth-degree patellar luxations can be problematic and reluxate. Complications in dogs have been associated with body weight. More complications are observed in dogs over 20 kg^37,38^. Her problem persisted as the dog previously had surgeries to cure the patellar luxation. We wanted to try an unconventional way to prevent the luxation. Because possible options such as condyle prothesis with normal anatomic condylar height, may not prevent the luxation. This may be due to excessive lateral retraction of the patella by the quadriceps muscles. The dog weighed 46 kg, which was a problem for prothesis due to the high amount of force passing through the condyle. Together with 3D scanning and 3D printing technologies, we created an overformed (extra-anatomic) implant with surgeon’s haptic experience. We combined surgeon’s hands with manufacturing steps to solve the problem with the overformed shape of the implant. The overformed implant was designed large enough to block the patella in trochlear sulcus. The implant succeeded in blocking the patella and there was no bone reaction. Compared with condyle replacement, knee replacement or trochlear sulcoplasty, surgeons did not perform any resection of the cartilage. The method was much less traumatic than traditional methods. However, there was a fistule on the surgical side from previous surgeries. This may have caused soft tissue inflammation. This caused lameness of the leg. In surgery, haptic experience is gained with hands and eyes. After many experiences, the correct shape, quantity and size can be understood by surgeon’s hands^39–42^. The correct size of the implant was made by the surgeon with this tactile information. It can not be designed with computer aided programs. Because every design made with the program will be relative and touching the implant will be superior to it. The implant did not form a prominent bulge after suturing the capsula. We did not observe any extension and flexion problems in the knee when it was controlled manually in the postoperative period. If there were any problems with the size of the implant, joint movements would not be possible due to the pressure inside the capsula. This means that the method can be useful for experienced surgeons. Three or four different size implants can be made to avoid unexpected problems.

If production is attempted in a hospital with 3D printer and CT, a patient-specific overformed polyamide implant can be made for surgery within a maximum of 2 days. The most time-consuming part is to convert CT data to STL format and create the implant with self-hardening paste. This production method requires collaboration between engineers, technicians and surgeons. Replica and implant may also have CAD program and printing errors. In order to prevent the surgeon from making a shaping error, the replica of the implant is made by using the paste on the replica of the patient’s original anatomical bone. The shape of the implant is not created with a computer program. The surgeon's understanding of the principles of the method and haptic experience will ultimately determine the success of the implant.

## Conclusion

Biological and biomechanical environment including the implant determines the results of the surgeries^28^. With all the factors affecting the region, we ensured that the patella remained in place. Further studies will demonstrate the effectiveness of the method. In addition, there may be difficulties in implant placement and prosthetic complications in tumor defect surgeries, critical zygomatic bone and vertebral fractures. These complications are caused by the different anatomy on the trauma side and the presence of vital vessels and nerves around it^43–46^. Overformed implantshapes may be more functional in some circumstances. We believe overformed anatomical implant design is promising in solving some of the challenges in implant surgeries by involving the surgeon's hands in rapid prototyping with surgeon’s haptic experience.

## Supplementary Information

Supplementary Information.

## Data Availability

All data generated or analysed during this study are included in this published article and its supplementary information files.
