# Supplementary material for: A new patient-specific overformed anatomical implant design method to reconstruct dysplastic femur trochlea
Source: Sci Rep. 2023 Feb 24;13:3204. doi: 10.1038/s41598-023-30341-4 (PMC9958018; doi:10.1038/s41598-023-30341-4)
Supplement: Supplementary file 1 — Supplementary Information. [file 41598_2023_30341_MOESM1_ESM.docx]

**Supplementary Data of ‘’** **A new Patient-specific Overformed Anatomical Implant Design Method to Reconstruct Dysplastic Femur Trochlea’’**

**Yetkin Öztürk * (1), Murat Ayazoğlu (2), Çağrı Öztürk (3), Atakan Arabacı (3), Nuri Solak (3), Serhat Özsoy (4)**

1. **Design workflow of overformed implant**

It is critical to change the computer tomography data to 3D printer data (stl format). After that defected bone can be printed with any polymer and the method can be applied on it. The workflow is described in supplementary figure 1.

**
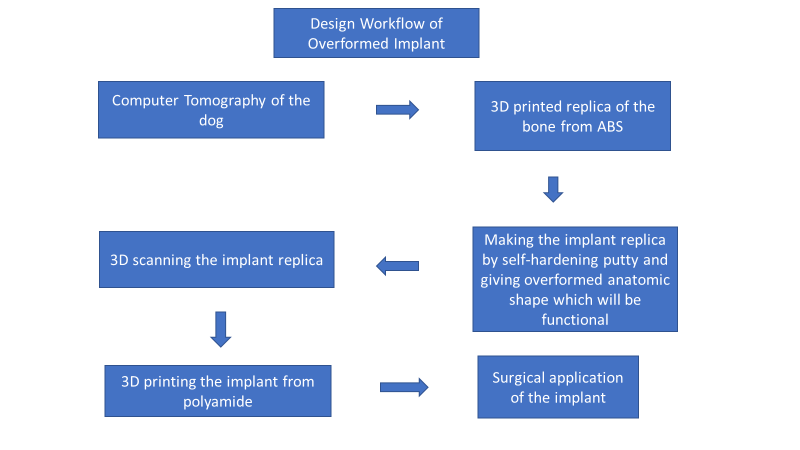
**

**Supplementary figure 1:** The workflow showing the production steps.

1. **Production of Implant**

Different sizes of implant replicas (green ones) were created on the replica of the bone. Many copies were printed with polyamide (white ones). Surgeon puts enough amount paste on the related part of the bone replica. Now, this is our workstation. Surgeon gives the shape and height of the implant which is irregular, nonanatomic, functional and has a volume which does not create a problem. Surgeons can only do it with experience, anatomical knowledge and haptic knowledge. The putty (supplementary figure 2) is made of two parts. When they are mixed a chemical reaction occurs and it hardens approximately in 30 or 40 minutes. After 20 minutes, every 5 minutes before hardening the putty must be moved slightly in order not to connect to the bone replica (the blue one). It is important to check its shape. There should be no space between the implant and the bone replica.

This method is applicable for all parts of the bones in the body. Author’s advice is when a problem can be solved with standard implants such as bone plates or biomedical implants, these can be used. However, extraordinary problems can be solved with patient-specific extra-anatomic implants. This implant method fully depends on the surgeon and the problem on the bone.


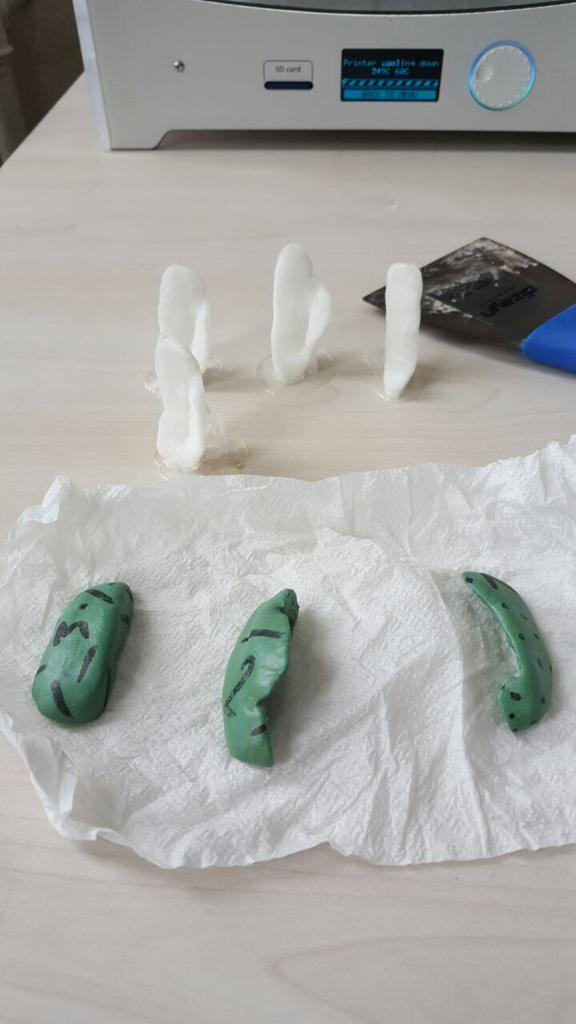


**Supplementary figure 2:** The green ones are hardened putty on the replica. White ones are printed according to hardened putty.

The implant comes out of the printer with its supports (supplementary figure 3). Supports were cut and protrusions on the entire surface were smoothed with sandpaper. The quality of the printer affects the quality of the shape of the implant. Testing of the implant on the replica is shown in supplementary figure 4.


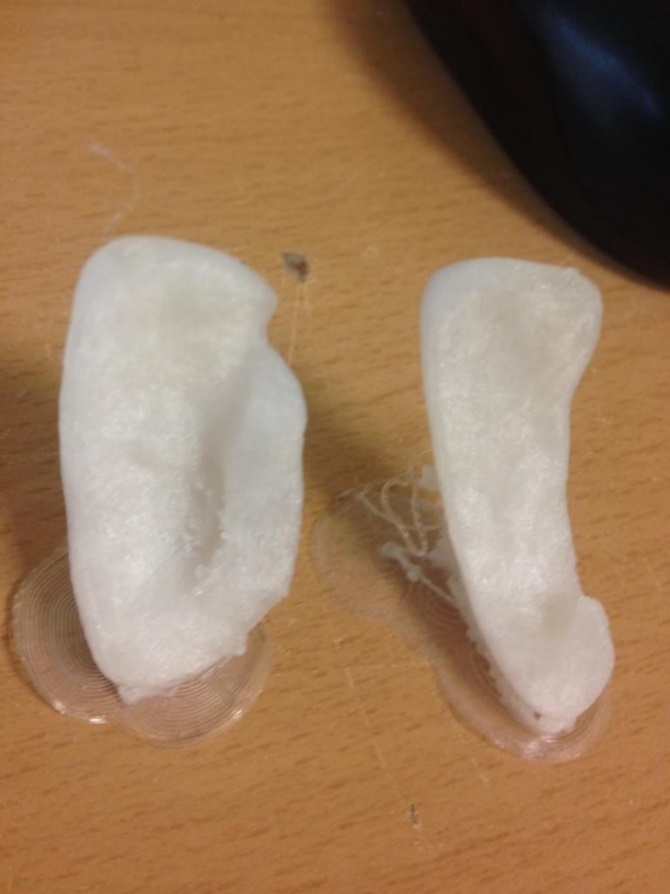


**Supplementary Figure 3:** Supports of the implant after printing can be seen.


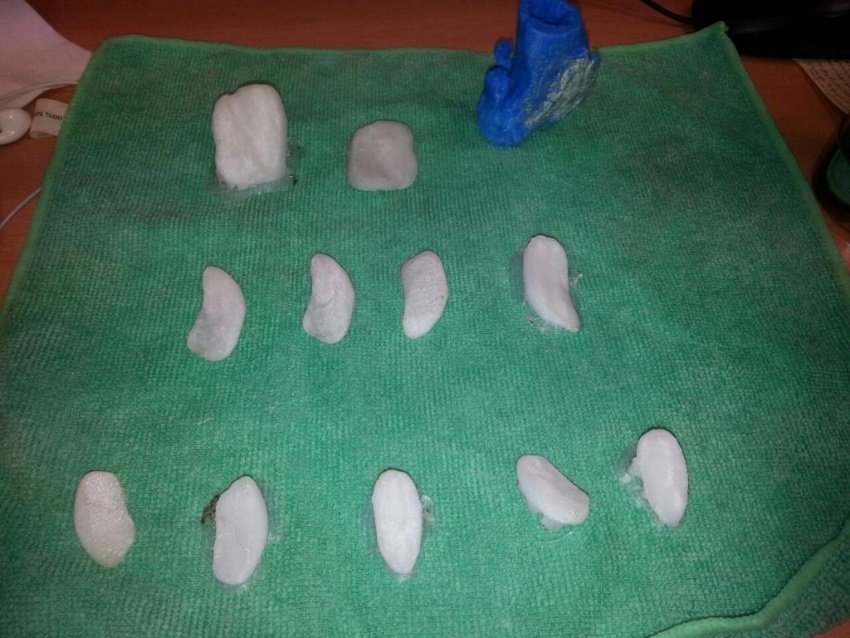


**Supplementary Figure 4:** All biocompatible polyamide implants (white ones) were tested on the bone replica (the blue one). After that, they were all sterilized.

1. **Sterilization control**

All the overformed implant samples were sterilized by autoclave at 121°C and 1.5 atm pressure for 20 minutes. After that, one implant was tested with yeast extract peptone dextrose. It was inserted in a falcon tube under laminar flow. It was incubated at 30°C and 150 rpm for 11 days. No turbidity was detected after 11 days of incubation of the implant in yeast extract peptone dextrose as it is shown in supplementary figure 5. The colour stayed the same.


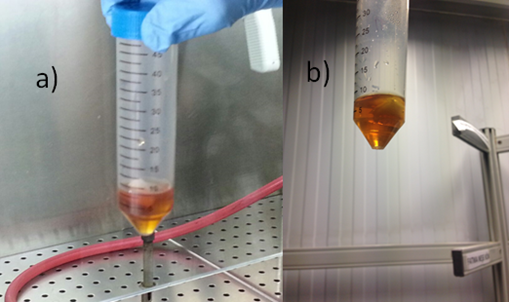


**Supplementary Figure 5:** A polyamide 3D printed implant sample incubated in yeast extract peptone dextrose (a) and after 11 days of incubation (b).
